# GNE Myopathy With Novel Mutations and Pronounced Paraspinal Muscle Atrophy

**DOI:** 10.3389/fneur.2018.00942

**Published:** 2018-11-08

**Authors:** Tyler Soule, Cecile Phan, Chris White, Lothar Resch, Atilano Lacson, Kristina Martens, Gerald Pfeffer

**Affiliations:** ^1^Hotchkiss Brain Institute, University of Calgary, Calgary, AB, Canada; ^2^Department of Medicine, University of Alberta, Edmonton, AB, Canada; ^3^Department of Clinical Neurosciences, University of Calgary, Calgary, AB, Canada; ^4^Department of Pathology and Laboratory Medicine, University of Calgary, Calgary, AB, Canada; ^5^Department of Laboratory Medicine and Pathology, University of Alberta, Edmonton, AB, Canada

**Keywords:** hereditary inclusion body myopathy, distal myopathy, GNE, genetics, muscle MRI, muscle pathology, paraspinal muscle atrophy

## Abstract

GNE myopathy is characterized by distal muscle weakness, and caused by recessive mutations in *GNE*. Its onset is characteristically in young adulthood, although a broad spectrum of onset age is known to exist. A large number of mutations in *GNE* are pathogenic and this clinical phenotype can be difficult to differentiate clinically from other late-onset myopathies. We describe two families with novel mutations in *GNE*, and describe their clinical and MRI features. We also describe the presence of striking paraspinal muscle involvement on MRI of the lumbar spine, which is an under-recognized feature of GNE myopathy.

## Introduction

GNE myopathy is an autosomal recessive disease with an onset usually in the twenties or thirties. Its characteristic onset is foot drop, which then progresses to further involvement of distal and proximal muscles and characteristically sparing the quadriceps muscles (1, 2). This disease is caused by mutations in *GNE*.

This condition was originally described as hereditary inclusion body myopathy in Jewish people of Persian descent, with a p.Met712Thr founder mutation. Since then, another founder mutation in Japanese patients was identified, which linked *GNE*-related diseases with the clinical descriptions of Nonaka myopathy and some cases of distal myopathy with rimmed vacuoles (1). There are now almost 150 known mutations associated with GNE myopathy, the vast majority of which are missense (2, 3).

We describe two new families with GNE myopathy who have two novel mutations in *GNE*, and describe neuroimaging features from the axial musculature in one patient, emphasizing another MRI finding that can raise clinical suspicion for this disorder.

## Methods

### Study participants

We identified 2 probands with novel *GNE* mutations from the practices of two of the authors (CP and CW). All study subjects provided written informed consent for research participation and publication of this case series. Research ethics board review was obtained from the University of Calgary Conjoint Health Research Ethics Board (REB15-2763).

### DNA and RNA extraction

Genomic DNA was extracted using QiAMP blood mini kit (Qiagen) according to manufacturer's protocol from EDTA whole blood tubes. RNA was extracted using a PAXgene blood RNA kit (PreAnalytix) from PAXgene RNA tubes. cDNA was synthesized using Superscript III (Invitrogen) with random hexamers according to manufacturer's protocol.

### Sanger sequencing

Sanger Sequencing was performed using custom primers with Primer3. PCR amplification was performed using custom primers and Qiagen Taq polymerase. Amplified products were purified using ExoI-SAP, chain termination sequencing using Bigdye (Applied Biosystems), on an ABI3130XL sequencer (Applied Biosystems). Data analysis was performed using Mutation Surveyor 5.0.1. Sequencing of cDNA (to identify alteration of the acceptor splice site at exon 8) was performed using primers specific for *GNE* exons 7–9, and the above Sanger sequencing methods. All primer sequences are available upon request. Sequence data were aligned to the following reference sequences: NM_001128227 and CCDS47965.

### RT-qPCR

Taqman probes for *GNE* were used according to standard protocols on a Quantstudio 3D digital droplet PCR system (Thermo Fisher) and analyzed using provided software.

## Results

### Clinical data

#### Proband 1

The patient was of Venezuelan origin and came to medical attention at age 34. She became aware of a foot drop at age 27. She was stable for 3 years, but began to fall during pregnancy. She then noticed weakness of her right leg. Her weakness has been slowly progressive since. At age 33 she noticed weakness of her proximal arms. She had never had diplopia, ptosis, dysphagia, dysarthria or respiratory symptoms. Myalgia and cramping were minimal. The cranial nerve assessment was normal with no evidence of weakness. She demonstrated a geographic pattern of weakness with weaker neck flexion and predominantly proximal weakness in the upper extremities. In the lower extremities there was proximal (hip flexion 2/5), intermediate (knee flexion 2/5), and distal (ankle dorsiflexion 0/5) weakness with complete sparing of the quadriceps and plantar flexors. Muscle bulk was decreased in weak muscles. There was mild scapular winging and hypermobility at the elbows, knees and wrists. Mild swan neck deformities were noted in the fingers. Nerve conduction studies were normal. EMG revealed features consistent with a myopathy. The CK was mildly elevated at 297 U/L. Her parents, sibling, and daughter were examined and found to have normal strength. There was no family history of neurologic disease but consanguinity was present in the parents (first cousins).

**Figure 1:**
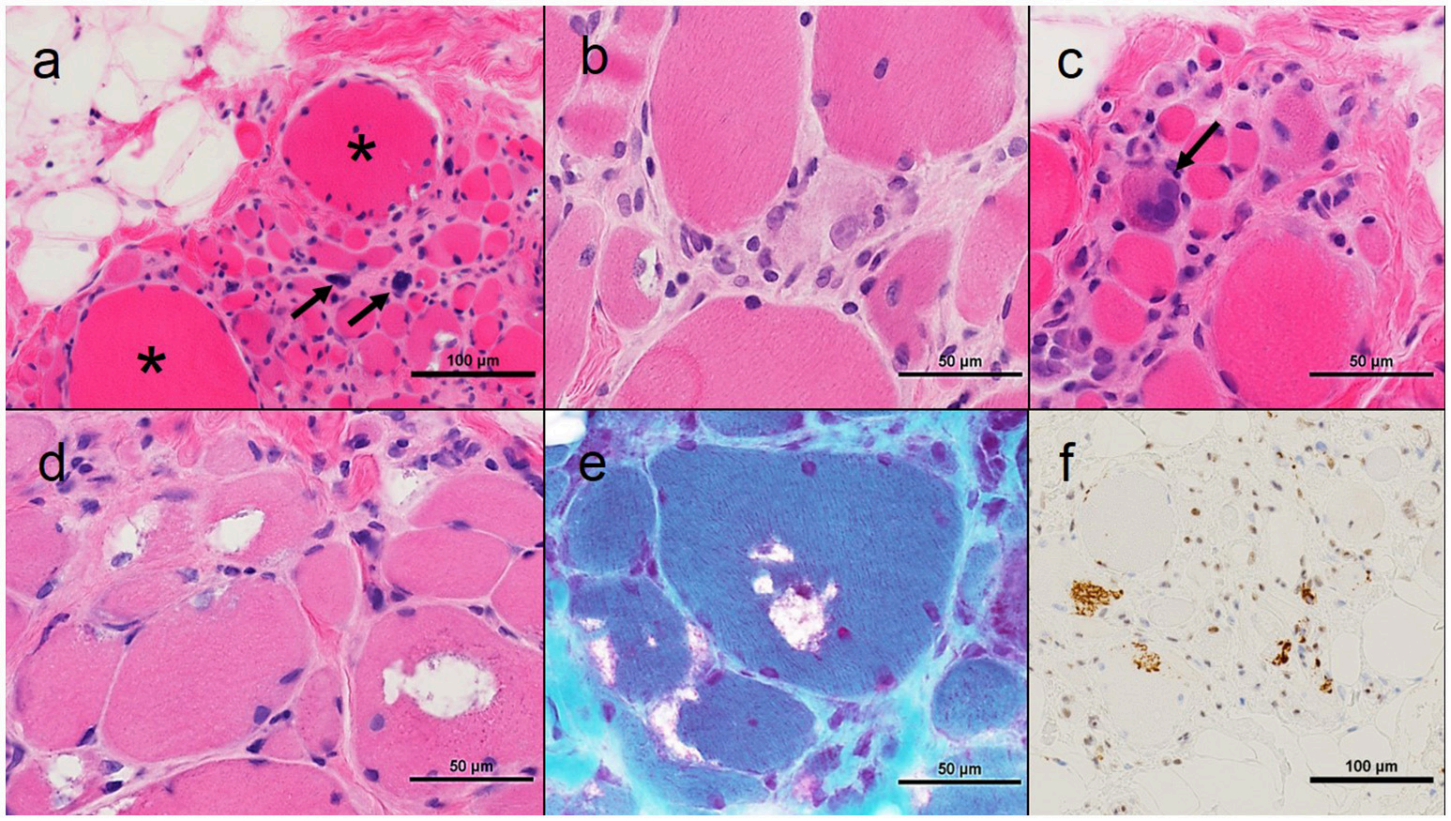
Muscle pathology from Proband 1. **(A)** Low power photomicrograph showing myofiber atrophy, hypertrophy (asterisks), pyknotic nuclear clumps (arrows) and fatty infiltration; **(B)** Myophagocytosis center of image; **(C)** Regenerating myofiber (arrow); **(D)** Rimmed vacuoles; **(E)** Modified muscle trichrome, rimmed vacuoles; **(F)** TDP-43 positive inclusions. All images haematoxylin and eosin unless otherwise stated; all cryostat sections except TDP-43 immunohistochemistry which is formalin fixed paraffin-embedded.

An open muscle biopsy of the right biceps was performed (Figure 1). This showed extensive myofiber atrophy with occasional nuclear bags and compensatory myofiber hypertrophy. This was accompanied by extensive endomysial fatty infiltration. Myophagocytosis and regenerating myofibers were present. Inflammation was present only in association with the necrotic myofibers. Rimmed vacuoles and TDP43-positive inclusions were identified in many myofibers.

#### Proband 2

**Figure 2:**
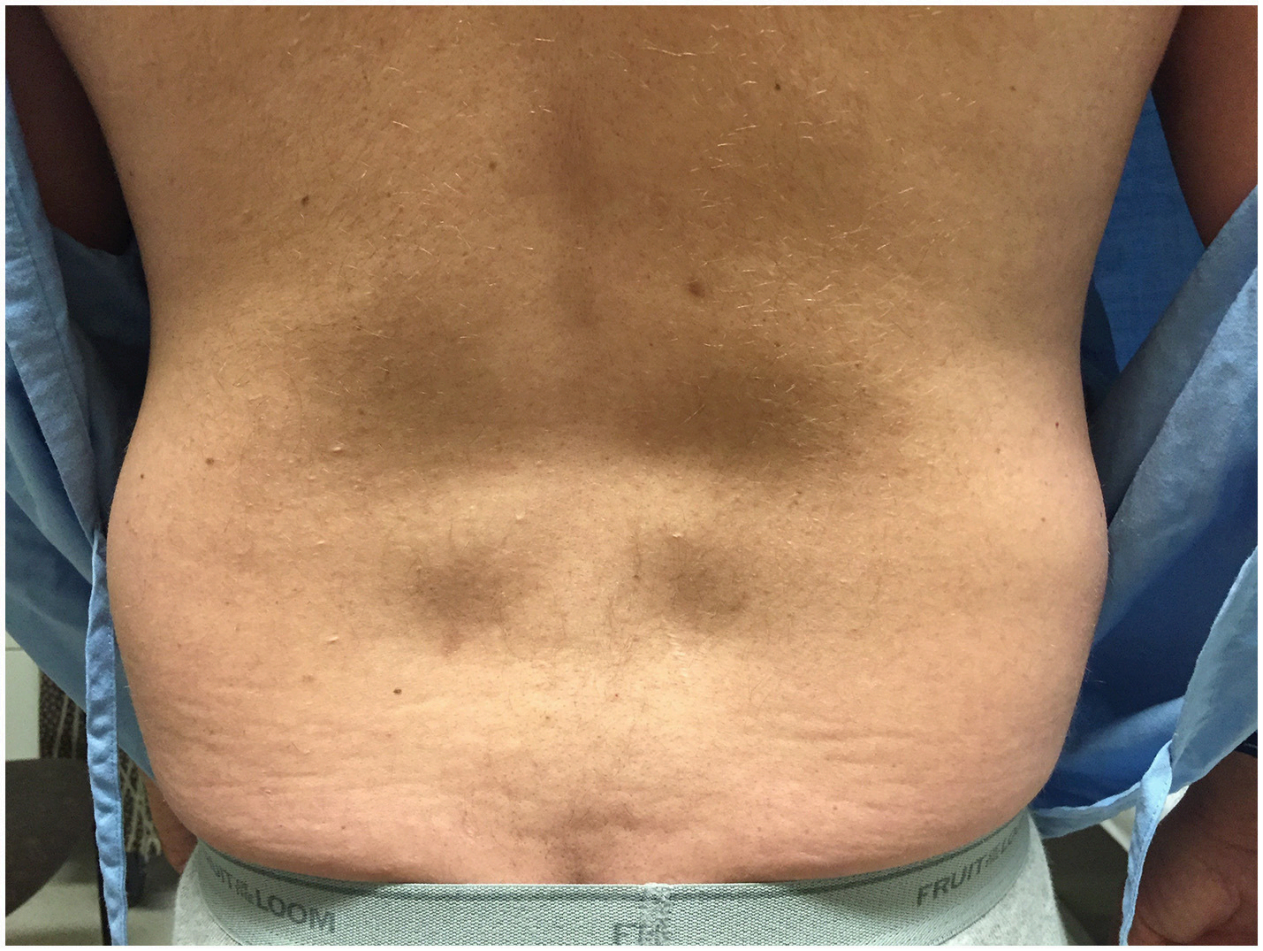
Paraspinal muscle atrophy in Proband 2. Image of Proband 2's back indicating severe lumbar paraspinal atrophy.

**Figure 3:**
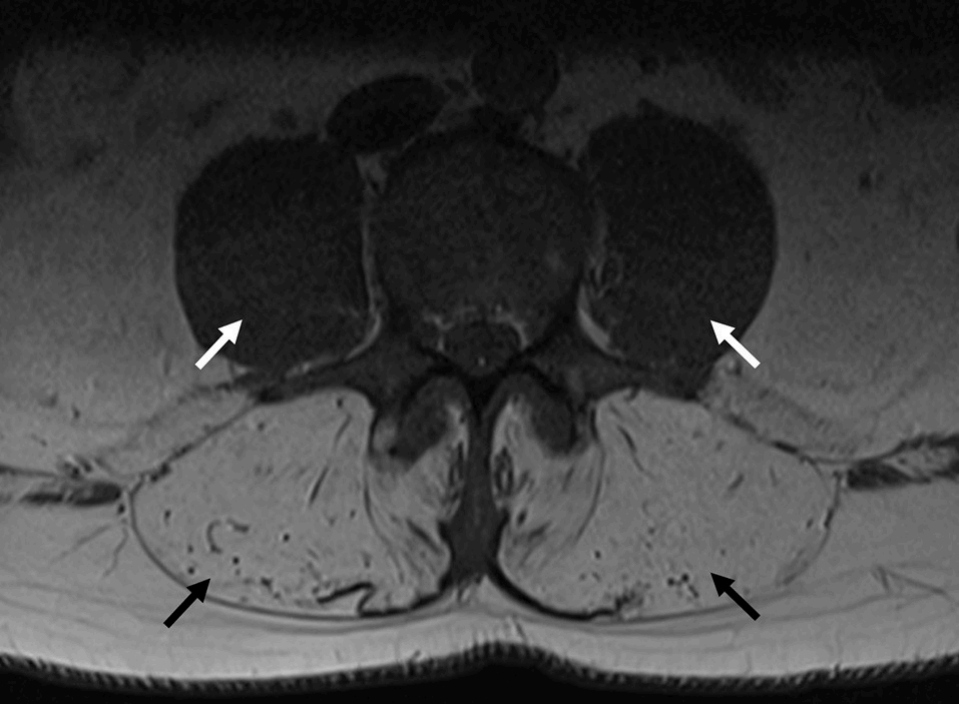
MRI of the lumbar spine from Proband 2. Transverse axial image of a T1 sequence in the lumbar spine, indicating severe paraspinal muscle atrophy (black arrows), with preservation of psoas muscles (white arrows).

The patient presented to clinic at age 45 with a history of progressive weakness and atrophy of his lower back and leg muscles. He first noticed symptoms 7 years prior with weakness of the lower back followed by gradual onset of bilateral foot drop over the past 2 years. He was formerly involved in multiple sports including ice hockey and football but was no longer able to engage in these activities. Walking up and down stairs and hills was particularly difficult. He did not have any symptoms in the upper extremities, facial, bulbar, respiratory or cardiac domains. He was on atorvastatin very briefly in the past, and stopped taking ezetimibe for several months. His parents were non-consanguineous. He had one brother who was asymptomatic, and one sister who was starting to show similar symptoms but much less severe. He had two children neither of whom manifested any symptoms. Physical examination revealed striking atrophy of the lumbar paraspinal muscles (Figure 2). There was no winging of scapula, calf hypertrophy, or contractures. There was atrophy of the anterior compartment of both legs with milder atrophy of the gastrocnemii bilaterally. Neck flexors, extensors, and muscles of the upper extremities were preserved except for mild weakness of biceps. The lower extremities were much more involved, especially the distal muscles. While hip flexors and extensors were 4/5 and 4+/5, respectively, ankle dorsiflexors and toe extensors were 1/5, and ankle inverters and everters were 1/5. Finger flexors, quadriceps, and gastrocnemius were spared. His gait was waddling with excessive lumbar lordosis. CK was elevated at 1,124 U/L. EMG of both tibialis anterior showed a mixed neurogenic and myopathic picture with moderately reduced recruitment rate but some of the units were very short, others were small and polyphasic. MRI of the lumbar spine revealed striking atrophy of paraspinal muscles, correlating with similar finding on examination, while pelvic and psoas muscles were spared (Figure 3).

A biopsy of the left vastus lateralis was performed. This showed skeletal muscle with scattered small angulated type I fibers consistent with neurogenic atrophy. No myopathic changes were seen (data not shown).

**Figure 4:**
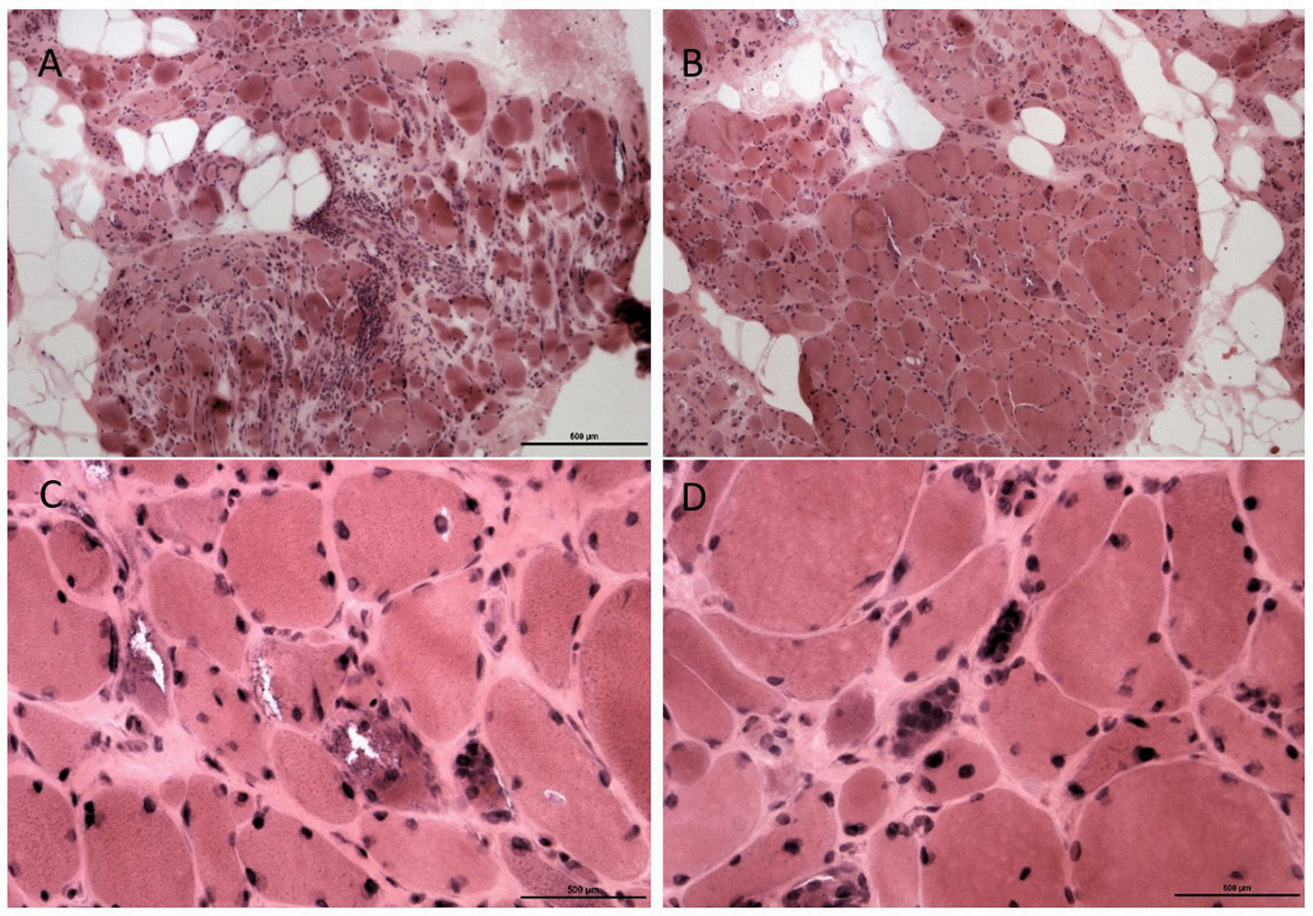
Muscle pathology from Proband 2, second muscle biopsy. Biopsy from the tibialis anterior showed marked myofiber atrophy and focal hypertrophy with fat replacement **(A)**. An adjacent fascicle shows similar but more moderate changes **(B)**, original magnification × 200. Several fibers show rimmed vacuoles **(C)**, with atrophic fibers containing collection of nuclei (nuclear bags, **D**), original magnification × 400.

A second biopsy of the tibialis anterior 1 year later (Figure 4) showed marked myopathic changes with focal endomysial fatty infiltration, perimysial and endomysial fibrosis, and variation in myofiber sizes into myopathic groups made up of small polygonal fibers with loss of sarcoplasmic details. There were also hypertrophic myofibers and myofibers with rimmed vacuoles. Internal nuclei were present along with nuclear bags. Focal deposits of ubiquitin and tau protein were seen in myopathic fibers. No congophilic amyloid deposits were seen. These sequential biopsies, a year apart, confirm the pathologic variation in involvement between the distal and proximal muscles in this case, which is typical for GNE-related myopathy.

### Sanger sequencing

Proband 1 had a homozygous missense variant c.1375G>A in exon 8, predicted to cause a simple amino acid substitution p.(Gly459Ser) within the kinase domain of GNE. Both parents were heterozygous for the same variant. The unaffected sibling was homozygous for the reference sequence at this position.

Proband 2 and his affected sister had compound heterozygous variants c.715G>A/p.(Asp239Asn) and c.1225G>T/p.(Asp409Tyr), which are both located in the epimerase domain of GNE. The proband's mother had only the c.1225G>T variant, indicating the two variants are on opposite alleles.

Sequencing of cDNA in Proband 1 was performed to determine whether alteration of splicing occurred given *in silico* predictions of altered splicing (c.1375G>A occurs in the first nucleotide of exon 8). PCR amplification and agarose gel electrophoresis identified a normal-sized product. Sequencing identified normal sequence order for exons 7–9 (data not shown). RT-qPCR for *GNE* transcript levels in Proband 1 overlapped with controls (data not shown).

## Discussion

We describe cases of GNE myopathy having characteristic clinical and myopathological features, providing data confirming pathogenicity for two novel mutations in *GNE*. The amino acids altered by these novel mutations are highly conserved.

The c.1375G>A variant in Proband 1 is a novel variant that has not been reported in the literature, is reported once (1/121,092) in the heterozygous state in ExAC (4), but is not present in dbSNP or other population databases (accessed April 5, 2018). *In silico* prediction tools (Mutation Taster, Polyphen 2, Human Splicing Finder, accessed April 5, 2018) predict the variant is pathogenic on the basis of this being a conserved amino acid and alteration of an acceptor splice site.

The c.1225G>T mutation from Proband 2 is well-described as a pathogenic founder mutation, particularly in individuals of Northern British descent (5). However, the c.715G>A variant is novel and does not have an entry in population databases, nor has it been reported as a pathogenic variant in published literature (accessed April 5, 2018).

Both of these variants should be considered as pathogenic based on the fact that (a) clinical and myopathologic characteristics of these cases are typical for the identified molecular defect, (b) missense mutations in these domains of GNE are typical genetic lesions associated with *GNE* myopathy (3), (c) variants are not present in controls (with the exception of c.1375G>A present in one person in heterozygous state), (d) family segregation in both cases provides supportive evidence, (e) the altered amino acid residues are highly conserved.

The case of Proband 1 emphasizes the importance of formally confirming splicing abnormalities which are predicted by *in silico* tools. Although the mutation is predicted to disrupt a consensus splice site, evidence for this could not be obtained from cDNA sequencing or transcript levels using RT-qPCR. The pathogenicity of this particular variant appears to be caused exclusively by its alteration of the amino acid sequence rather than by altered splicing.

Another noteworthy feature of Proband 1's case is her significant clinical deterioration during pregnancy. This has been described in prior cases and is likely related to increased sialic acid requirements during pregnancy (6–8). This clinical feature may be considered as a unique component of the GNE myopathy phenotype, and deterioration of genetic myopathy during pregnancy has also been described in mitochondrial syndromes (9–11), which can be distinguished from GNE myopathy as a characteristically multisystem disorder.

The case of Proband 2 emphasizes the value of assessing paraspinal muscles on MRI, since the involvement is a striking feature in this case. This abnormality has been observed in a subset of previously reported cases (12), often in conjunction with atrophic abdominal muscles (13). However, it should be noted that previously described cases with paraspinal muscle involvement had advanced disease, while the case of Proband 2 had this finding as an early and prominent feature of his clinical presentation. This finding is not mentioned in expert reviews of this condition (14, 15), perhaps due to lack of ascertainment or awareness of this abnormality. However, we emphasize this finding for its utility in directing genetic investigation and in genotype-phenotype correlation. Early axial muscle involvement was also reported from one series of GNE myopathy patients, in which one patient had developed early respiratory failure whilst still ambulant (16).

Another interesting aspect of genotype-phenotype correlation in GNE myopathy is the finding that homozygous kinase domain mutations are associated with a more severe phenotype than compound heterozygous epimerase/kinase mutations (17). In these reported cases, Proband 1 has homozygous kinase domain mutations, and has a more severe phenotype, with earlier age of onset and more severe weakness, compared with Proband 2.

Further work could have provided additional evidence supporting the pathogenicity of these variants. *GNE*, along with *GFPT1* and *PGM3*, are associated with three distinct genetic disorders resulting from defects in the hexosamine and sialic acid pathway. These disorders have in common that an absence of precursors in glycosylation pathways cause tissue-specific disorders of glycosylation. GNE is responsible for production of cytidine-5′-monophospho-N-acetylneuraminic acid which is of greatest clinical importance in muscle tissue, resulting in late-onset distal myopathy (18). In the case of GNE myopathy, the biochemical defect can be measured directly in muscle tissue as has been reported previously (19, 20). Unfortunately, this was not possible for the described cases because additional muscle tissue was not available for analysis.

Muscle MRI is a modality that has demonstrated utility in a number of genetic myopathies and can provide added value in correlation with clinical phenotype, genetic testing and muscle pathology (21), and additionally has value in longitudinal studies and clinical trials (22). In GNE myopathy, MRI of the lower extremity muscles typically demonstrates severe and early involvement of the biceps femoris short head (12). Other involved muscles include gluteus minimus, tibialis anterior, extensor hallucis and digitorum longus, soleus and gastrocnemius medialis, with characteristic sparing of vastus lateralis (12).

The differential diagnosis for cases with this clinical phenotype includes a number of distal myopathies presenting with pronounced tibialis anterior weakness in adulthood. Tibial muscular dystrophy is caused by mutations in the M-line region of *TTN* and is differentiated from GNE myopathy by its very late onset, mild phenotype, and autosomal dominant inheritance pattern (23). Hereditary myopathy with early respiratory failure is caused by mutations in the 119th fibronectin-3 domain of TTN (24–26) and is differentiated from GNE myopathy because of its very early respiratory involvement and autosomal dominant inheritance. Laing distal myopathy is caused by dominant mutations in *MYH7* and has several similarities with GNE myopathy including the early tibialis anterior involvement and axial muscle involvement (27). However, the prominent involvement of hand extensors in this condition is not typical of GNE myopathy and not present in the described cases. Welander myopathy can have significant involvement of distal foot and ankle extensors however the most early and prominent feature in this condition is typically finger extensor weakness, which is not present in this case (28). Myofibrillar myopathies may present with early tibialis anterior involvement and can present in adulthood, although phenotypic features that differ from this case include the absence of cardiomyopathy, absence of myofibrillar pathology, and the inheritance pattern (29).

## Author contributions

TS and KM collection and interpretation of laboratory data, CP and CW collection and interpretation of clinical data, LR and AL collection and interpretation of pathology data, TS, CP, CW, LR, AL, and GP manuscript authorship, KM manuscript revision for intellectual content, GP supervision of laboratory studies.

### Conflict of interest statement

The authors declare that the research was conducted in the absence of any commercial or financial relationships that could be construed as a potential conflict of interest.
